# Left-right symmetry breaking: learning from the chicken

**DOI:** 10.3389/fcell.2025.1672263

**Published:** 2025-09-24

**Authors:** Tobias Karl Pieper, Nikoloz Tsikolia

**Affiliations:** Department of Anatomy and Cell Biology, University Medical Center Göttingen, Göttingen, Germany

**Keywords:** left-right symmetry breaking, gastrulation, chirality, vertebrate embryo, cytoskeleton

## Abstract

Morphological left-right asymmetry of visceral organs in most cases reveals a typical arrangement. This implies directed symmetry breaking which is suggested to be based on the existence of structural chirality. At early developmental stages many vertebrate model organisms display so-called leftward flow of extracellular fluid which is based on the unidirectional rotation of chiral cilia. Cytoskeletal chirality has been shown to contribute to the left-right asymmetry of invertebrates including *Caenorhabditis elegans* and *Drosophila melanogaster*. The mechanisms of left-right symmetry breaking in vertebrates without ciliary flow remain mysterious. Here, we present our perspective on left-right patterning and symmetry breaking in the chick within a broader context.

## Meeting point nodal

Left-right asymmetry of viscera is a fundamental anatomical feature of vertebrates and many invertebrates (Blum and Ott, 2018). Morphological asymmetry is manifested both in bilateral asymmetry or handedness of organs and their asymmetric position and was shown to be preceded by asymmetric gene expression and asymmetrical molecular signaling (referred to here as *left-right patterning*). Expression of TGF-beta member Nodal in the left lateral plate mesoderm is a common denominator of early left-right molecular patterning in studied vertebrates (Duboc and Lepage, 2008; Grande and Patel, 2009). Well-studied effector of asymmetric Nodal activity is the transcription factor Pitx2, which has been shown to be directly involved in asymmetric morphogenesis of the heart and the gut (Campione et al., 1999; Lin et al., 1999). Interestingly, the lateral plate mesoderm is involved in the morphogenesis of asymmetrical organs contributing to heart and connective tissues of the gut. Nodal-Pitx2 module was also reported to be involved in asymmetrical development of amphioxus, snails and sea urchins. Asymmetrical gene expression, however, is preceded by a symmetrical state, displaying either bilateral absence or bilateral presence of gene expression or molecular activity. Assuming original bilateral symmetry of a developing embryo, asymmetric gene expression is preceded by a process described as initial symmetry breaking which is a transition to a state with higher symmetry.

## The (chiral) form is the cause

The original idea proposed by Pierre Curie suggested that the symmetry elements in the cause must be found in their effects and asymmetries in the effect are derived from asymmetries in the causes (Curie, 1894). Further development of the symmetry breaking concept led to distinction between explicit symmetry breaking, which follows the above described definition and spontaneous symmetry breaking (Earman, 2004) where the outcomes are distributed equally (outcomes are randomized). An important feature of animal left-right symmetry breaking, however, is its directionality: the resulted sidedness after the symmetry breaking is not random. Hence, the initial animal left-right symmetry breaking cannot be described as spontaneous. This suggests underlying structural molecular asymmetry which may be based on chirality as known in chemistry. Wolpert and Brown (Brown and Wolpert, 1990) proposed the existence of a chiral molecular determinant denominated as F-molecule which generates initial asymmetry subsequently translated at cellular and organismal level. Afzelius reported a correlation between disturbed laterality and abnormal cilia (Afzelius, 1976): ultrastructural examination of immotile sperm in 4 patients with impaired mucociliary transport and associated recurrent bronchitis revealed the absence of dynein arms in the cilia with three out of four patients also showing situs inversus totalis. The association of bronchiectasis accompanied by sinusitis with situs inversus was already described by Manes Kartagener (Kartagener and Stucki, 1962). Afzelius proposed that the cilia motility in embryonic tissues critically contributes to the right-left asymmetries of adult organisms (Afzelius, 1976). As cilia reveal structural chirality they fulfill requirements of the hypothetic F-molecule. Indeed, shortly after the proposed role of the hypothetical F-molecule, motile cilia were shown to be present in early mouse embryos during gastrula and early somitogenesis: the ventral surface of epithelialized midline cells assigned to the notochord and node possess long motile monocilia (Sulik et al., 1994). Based on results of Afzelius and observation that the comparable developmental stage in rat embryos is critical for the establishment of right-left asymmetry (Fujinaga and Baden, 1991), Sulik and co-workers suggested a causal contribution of motile monocilia to left-right symmetry breaking (Sulik et al., 1994). Screening of mouse strains with complete inverted viscera revealed mutations in cilia-associated axonemal heavy-chain dynein (Supp et al., 1997). Similarly, it was shown that mice depleted for kinesin KIF3B do not form monocilia and reveal randomized left-right asymmetry (Nonaka et al., 1998). Furthermore, in WT embryos corresponding cilia undergo rotation accompanied by leftward fluid flow as shown by video microscopy of ventral surface of embryos cultured in a medium with fluorescent beads. The experimental reversal of flow direction led to the formation of situs inversus while an artificially generated flow in embryos with defective cilia “rescued” the phenotype (Nonaka et al., 2002).

## The direction (of flow) decides where it becomes the left side

**FIGURE 1:**
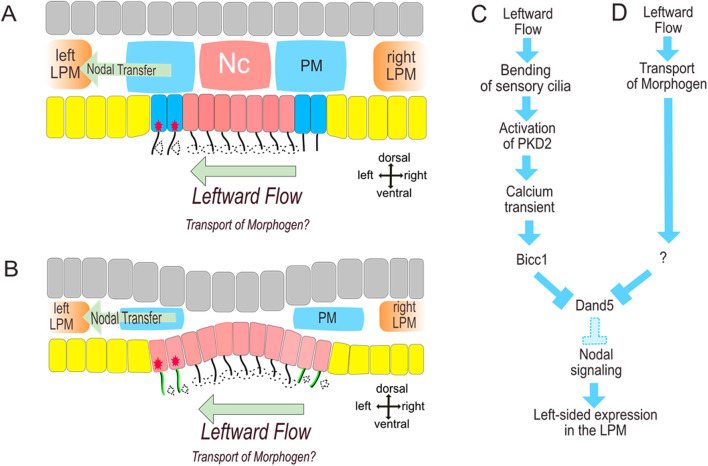
Left-Right-Organizer and Symmetry breaking in *Xenopus* **(A)** and Mouse **(B)**. **(A,B)** morphology, **(C)** important steps of two-cilia model, **(D)** proposed transport of morphogens. Clockwise rotation of monocilia in the midline of gastrocoel roof plate **(A)** or posterior notochord **(B)** creates leftward flow which causes deflection of sensory cilia formed by superficial somitic progenitor cells. Deflection mediates Calcium transients (red stars) and further processes leading to left-sided nodal activation in the paraxial and later in the lateral plate mesoderm **(C)**. Yellow: endoderm, grey: ectoderm, red: axial mesoderm, dark blue: superficial somitic mesoderm, blue: deep somitic mesoderm, LPM: lateral plate mesoderm. PKD localization (green) in the medial (dorsal) segment in mouse immotile cilia **(B)** ensures left-sided activation despite the deflection of the right-sided sensory cilia to the left (dashed arrows at the right side of the LRO).

Leftward flow was observed in frog *Xenopus laevis*, zebrafish and mouse while morphological signs of a ciliated organizer were reported for many further but not for all studied vertebrates (Schroder et al., 2016; Blum et al., 2007). Successful symmetry breaking requires a temporary formation of a specific “organ”: the ciliary leftward flow is located at the ventral surface of specialized structure called left-right organizer (LRO, cf. Figure 1). Crucially, symmetry breaking by leftward flow is followed by subsequent activation of nodal signaling at the left-side (Schweickert et al., 2010; Oki et al., 2009). Two mechanisms were proposed to explain how leftward flow caused left-right patterning. Initial proposal suggested leftward transport of morphogens (Nonaka et al., 1998) while later studies suggested existence of sensory immotile cilia (McGrath et al., 2003; Tabin and Vogan, 2003). Indeed, recent studies in mouse and zebrafish embryo strongly support the view that deflection of immotile cilia by flow which lead to polycystin-2 (Pkd2) channel mediated activation of intraciliary Calcium transients (Katoh et al., 2023; Djenoune et al., 2023). How asymmetrical Calcium transients influence left-right patterning is to be investigated in more detail (Mizuno et al., 2020). Particularly, it has been suggested that Calcium transients activate RNA binding protein Bicc1 which in turn suppresses the translation of nodal antagonist *dand5* mRNA (Maerker et al., 2021). The LRO is formed by axial and (in *Xenopus*) paraxial mesoderm progenitors which form the gastrocoel roof plate in amphibian (Shook et al., 2004; Blum et al., 2009), the posterior notochord in mouse and rabbit (Schroder et al., 2016; Blum et al., 2007) or the Kupffer’s vesicle in zebrafish embryos (Essner et al., 2005). Function of LRO requires the correct domain architecture and differentiation. The effectively directed flow was suggested to be caused by posterior position and structural features of the motile cilia (Nonaka et al., 2005). Hence, the correct position of cilia is a function of established planar cell polarity along anterior-posterior axis (Antic et al., 2010; Hashimoto and Hamada, 2010; Hashimoto et al., 2010; Song et al., 2010; May-Simera et al., 2010; Borovina et al., 2010). In *Xenopus* and mouse embryo LRO includes spatially segregated domains characterized by gene expression and types of cilia (Schweickert et al., 2010; Blum et al., 2009; Petri et al., 2024). Particularly, lateral domains which form at both sides immotile cilia bilaterally express *nodal*, its antagonist *dand5* and in *Xenopus* somitic marker *myoD* (Schweickert et al., 2010; Petri et al., 2024; Tingler et al., 2022). Importantly, to create the appropriate flow and detect it, ciliated cells should be transiently faced towards a cavity of the LRO whereas after the symmetry breaking event, the surface is covered by endodermal cells. Abnormal development of LRO with a LRO covered by endoderm prevents correct symmetry breaking in *Xenopus* (Schneider et al., 2019; Petri et al., 2022). Recent observations indicate involvement of morphogen transport in left-right symmetry breaking (Ott et al., 2025; Lee et al., 2024) suggesting a complex and possibly double-secured mechanism. Flow-mediated transport of PKD to the left of the mouse LRO has been suggested to contribute to left-right symmetry breaking in the mouse embryo (Tanaka et al., 2023). It must be mentioned that further, earlier asymmetries have been observed in *Xenopus* and mechanisms based on these have been discussed (Onjiko et al., 2021; Onjiko et al., 2016; Vandenberg and Levin, 2013), which still have to be integrated into the existing models.

## There is another way

Leftward flow has been suggested to be required for directed LR symmetry breaking in many vertebrate models (Blum and Ott, 2018; Hamada, 2020; Blum et al., 2014) and is one of the most fascinating observations in developmental biology. However, structural elements required for leftward flow are absent in several model organisms. Structural units required for ciliary flow were not detected in chick and pig embryos where the ventral surface of axial mesoderm was shown to be covered by endoderm and subchordal mesoderm (Manner, 2001; Gros et al., 2009) while analysed non-avian reptilian do not form motile cilia (Kajikawa et al., 2020; Shylo et al., 2023). Furthermore, in the chick, the first signs of left-right asymmetry occur at late gastrula stage and include the morphological asymmetry of the node and asymmetric leftward cell movements around the node (Dathe et al., 2002; Cui et al., 2009). These observations also match the asymmetric gene expression in the chick node prior to asymmetry of *nodal* expression in the lateral plate mesoderm: *sonic hedgehog* morphogen (shh) was shown to be expressed asymmetrically in the node of notochord stage (stage 5) chick embryo (Levin et al., 1995; Pagan-Westphal and Tabin, 1998) while this asymmetry was suggested to cause left-sided *nodal* expression at somitogenesis stages. Further analysis of chicken node suggested asymmetrical morphology of the node itself prior to asymmetric gene expression (Dathe et al., 2002).

We suggest that the chick primitive node plays the role of the LRO. In the following we explain the corresponding developmental context.

## This is the way (of the chicken)

Gastrulation in the chick starts with the formation of the primitive streak in the posterior area of circle-shaped bilayered embryonic disc (stage 1). Cell intercalation in the posterior epiblast has been suggested as a cellular mechanism of primitive streak formation. Intercalation was shown to be PCP-dependent (Voiculescu et al., 2007) and/or be based on Myosin-II based contractions of epiblast cell groups (Rozbicki et al., 2015). Cell division in the anterior pole of the embryo accounts for the tissue fluidity that is necessary for primitive streak formation (Firmino et al., 2016) while the tensile ring at the margin of the *area pellucida* is required for tissue flow during primitive streak formation (Saadaoui et al., 2020). Primitive streak undergoes elongation which is accompanied by bilateral flow of cells of dorsal (epiblast) layer towards the streak. The node which gives rise to the axial mesoderm emerges at the anterior tip of the streak and starts to form the prechordal mesoderm (Stern and Stern, 2004). The maximum of elongation of the primitive streak is followed by the beginning of notochord formation and concomitant shortening of the streak also referred to as streak regression. Global cell movements towards the emerging streak reveal left-right asymmetry, even if the functional significance of this asymmetry remains to be tested (Asai et al., 2025). The first morphological asymmetry arises in the node at the beginning of notochord formation: dorsal views reveal that the right edge of the node is thicker than the left edge while sections demonstrate that this difference is due to mesodermal density at the right side below the epithelial epiblast (Tsikolia et al., 2012). Asymmetric position of density is already detectable prior to the emergence of the notochord. Emergence of the asymmetric notochord is preceded by asymmetrical counterclockwise cell movements within the node (cf. Supplementary Figure). It was suggested that the progressive asymmetry of *sonic hedgehog* expression is due to displacement of *shh* domain to the left-side while pharmacological evidence indicates that node rotation requires actomyosin contractility and activity of ATP4a proton pump (Gros et al., 2009). Moreover, N-Cadherin is involved in termination of node rotation (Mendes et al., 2014). Further analysis however, revealed, that paraxial asymmetrical expression of *nodal* is initiated at the beginning of notochord formation while *shh* is still expressed at both sides of the node (Tsikolia et al., 2012) hence indicating that *nodal* asymmetry is not a direct effect of node and *shh* domain rotation. Asymmetry of the node and asymmetric *shh* expression increase concomitant with node regression during stages 5 and 6. Surprisingly *shh*-expression at these stages is confined to the epiblast of the left node shoulder, to the floor plate, to the prechordal mesoderm but not to the posterior notochord (Kremnyov et al., 2018) challenging the widely assumed induction of floor plate by the notochord.

**FIGURE 2:**
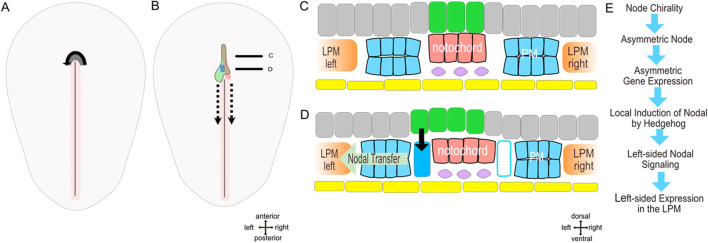
**(A–C)** model of molecular left-right patterning of early axial asymmetry in the chick (stage 5-). **(A,B)** dorsal views embryos prior (stage 4) and after the beginning (stage 5-) of notochord formation. **(C,D)** schematic sections through anterior and posterior notochord. **(E)** succession of events. Yellow–endoderm/hypoblast, pink–notochord, grey–epiblast, green–prospective floor plate with *shh* domain, dark blue–parachordal nodal domain, blue–parachordal mesoderm, rose–primitive streak with primitive groove, violet–subchordal mesoderm, dashed arrow–node translocation during regression, continuous arrow–nodal induction.

Morphological analysis of the node and notochord reveals the continuity of the notochord with the right node shoulder. Assuming that notochord progenitor cells are generated within the node this continuity suggests an asymmetrical notochord formation. This proposal is supported by analysis of notochordal marker expression particularly *noggin* (Otto et al., 2014) and *brachyury*. The origin of the notochord from the right side of the node results in positioning of the *shh* expressing floor plate to the left of the notochord and immediately above the paraxial *nodal* expressing area. This spatial proximity suggests a local induction of the paraxial *nodal* domain by hedgehog signaling activated by shh ligand secreted from the floor plate. Indeed, the inhibition of hedgehog signaling leads to an absent paraxial *nodal* expression (Otto et al., 2014) while ectopic hedgehog activation (Negretti et al., 2022) causes bilateral *nodal* expression. This data strongly suggests that secreted hedgehog is both necessary and sufficient for paraxial *nodal* induction in the chick while the asymmetry of *nodal* is due to asymmetry of the notochord formation (Figure 2). Interestingly, prior to asymmetric morphogenesis the node revealed matrix-filled spaces which may be involved in regulation of molecular activity (Pieper et al., 2020).

## Cytoskeleton: hidden egg?

What are the mechanisms leading to the asymmetric morphogenesis of the avian node? Leftward rotation of the node is a robust morphogenetic event and indicates intrinsic tissue chirality which in turn has been suggested to have roots in the cytoskeletal organisation particularly in chiral features of the cytoskeletal constituents such as microtubules or actin (Wan and Vunjak-Novakovic, 2011; Xu et al., 2007; Yamanaka and Kondo, 2015). Indeed, cytoskeletal asymmetries were shown to be involved in left-right symmetry breaking during development of *C. elegans* which reveals chiral cortical flow related to contractility of actomyosin (Naganathan et al., 2014) and regulated by RhoA and Cyk1/Formin (Middelkoop et al., 2021; Pimpale et al., 2020). Chiral development of snails is also controlled by formins dia1 and dia2 (Abe and Kuroda, 2019) which regulate actin nucleation and polymerisation while further actin nucleator DAAM (Chougule et al., 2020) together with unconventional myosin ID (Myo1D) are involved in left-right asymmetry in *Drosophila melanogaster* (Coutelis et al., 2008; Speder et al., 2006). Similar to other members of Myosin I family, Myosin ID was shown to link actin with membrane lipids (McAlpine et al., 2018) and it has been suggested that the role of Myosin ID as a chiral determinant is due to its chiral interaction with actin (Juan et al., 2018).

## What's next?

It has been proposed that cellular-scale chirality arises from chiral structures by interaction within “chirality modules” particularly between actin filaments and formin dimers, whereas the understanding of transition to tissue chirality remains challenging (Tsikolia et al., 2025).

How does the cellular chirality contribute to left-right symmetry breaking in the chick? At elongated streak stage only the node tissue displays chiral behaviour which manifests itself during a short period of time. This indicates that the supracellular manifestation of chirality is a subject of regulation. The node which is equivalent of Spemann’s organizer (Stern and Stern, 2004) undergoes different stages: it emerges in the area expressing organizer genes, contributes to the prechordal mesoderm, endoderm, medial paraxial and axial mesoderm. Node rotation takes place after migration of the prechordal mesoderm and clustering of axial mesoderm progenitors in the node mesoderm (Tsikolia et al., 2012). These events may stimulate activation of tissue chirality in the node area. Whether this activation is due to specific molecular pathway or structural constraint has to be investigated in the next step.

## Supplementary material

SUPPLEMENTARY FIGURE Asymmetrical node morphogenesis in the chick embryo **(A–C)** scanning electron microscopy of the node prior to notochord formation (stage 4: **A,B**) and at the early notochord stage (stage 5, **C,D**) embryos. **(B)** schematic view of asymmetrical node (green) displacement (as reported by Gros et al., 2009 and Cui et al., 2009), **(C)**– emergence of thicker right node shoulder (rs). **(D)** floor plate (green) and notochord (red) domains indicated at the surface. A modified from Figure 1F (Tsikolia et al., 2012) with permission.

## References

[B1] Abe M. Kuroda R. (2019). The development of CRISPR for a mollusc establishes the formin Lsdia1 as the long-sought gene for snail dextral/sinistral coiling. Development 146, dev175976. 10.1242/dev.175976 31088796

[B2] Afzelius B. A. (1976). A human syndrome caused by immotile cilia. Science 193, 317–319. 10.1126/science.1084576 1084576

[B3] Antic D. Stubbs J. L. Suyama K. Kintner C. Scott M. P. Axelrod J. D. (2010). Planar cell polarity enables posterior localization of nodal cilia and left-right axis determination during mouse and xenopus embryogenesis. PLoS One 5, e8999. 10.1371/journal.pone.0008999 20126399 PMC2814853

[B4] Asai R. Sinha S. Prakash V. N. Mikawa T. (2025). Bilateral cellular flows display asymmetry prior to left-right organizer formation in amniote gastrulation. Proc. Natl. Acad. Sci. U. S. A. 122, e2414860122. 10.1073/pnas.2414860122 39899727 PMC11831138

[B5] Blum M. Ott T. (2018). Animal left-right asymmetry. Curr. Biol. 28, R301–R304. 10.1016/j.cub.2018.02.073 29614284

[B6] Blum M. Andre P. Muders K. Schweickert A. Fischer A. Bitzer E. (2007). Ciliation and gene expression distinguish between node and posterior notochord in the Mammalian embryo. Differentiation 75, 133–146. 10.1111/j.1432-0436.2006.00124.x 17316383

[B7] Blum M. Beyer T. Weber T. Vick P. Andre P. Bitzer E. (2009). Xenopus, an ideal model system to study vertebrate left-right asymmetry. Dev. Dyn. 238, 1215–1225. 10.1002/dvdy.21855 19208433

[B8] Blum M. Feistel K. Thumberger T. Schweickert A. (2014). The evolution and conservation of left-right patterning mechanisms. Development 141, 1603–1613. 10.1242/dev.100560 24715452

[B9] Borovina A. Superina S. Voskas D. Ciruna B. (2010). Vangl2 directs the posterior tilting and asymmetric localization of motile primary cilia. Nat. Cell. Biol. 12, 407–412. 10.1038/ncb2042 20305649

[B10] Brown N. A. Wolpert L. (1990). The development of handedness in left/right asymmetry. Development 109, 1–9. 10.1242/dev.109.1.1 2209459

[B11] Campione M. Steinbeisser H. Schweickert A. Deissler K. van Bebber F. Lowe L. A. (1999). The homeobox gene Pitx2: mediator of asymmetric left-right signaling in vertebrate heart and gut looping. Development 126, 1225–1234. 10.1242/dev.126.6.1225 10021341

[B12] Chougule A. Lapraz F. Foldi I. Cerezo D. Mihaly J. Noselli S. (2020). The drosophila actin nucleator DAAM is essential for left-right asymmetry. PLoS Genet. 16, e1008758. 10.1371/journal.pgen.1008758 32324733 PMC7200016

[B13] Coutelis J. B. Petzoldt A. G. Speder P. Suzanne M. Noselli S. (2008). Left-right asymmetry in drosophila. Semin. Cell. Dev. Biol. 19, 252–262. 10.1016/j.semcdb.2008.01.006 18328746

[B14] Cui C. Little C. D. Rongish B. J. (2009). Rotation of organizer tissue contributes to left-right asymmetry. Anat. Rec. Hob. 292, 557–561. 10.1002/ar.20872 19301278 PMC2714534

[B15] Curie P. (1894). Sur la symmetrie des phenomenes physiques: symmetrie d'un champ electrique et d'un champ magnetique. J. de Physique 3 (3), 393–415. 10.1051/jphystap:018940030039300

[B16] Dathe V. Gamel A. Manner J. Brand-Saberi B. Christ B. (2002). Morphological left-right asymmetry of Hensen's node precedes the asymmetric expression of shh and Fgf8 in the chick embryo. Anat. Embryol. Berl. 205, 343–354. 10.1007/s00429-002-0269-2 12382138

[B17] Djenoune L. Mahamdeh M. Truong T. V. Nguyen C. T. Fraser S. E. Brueckner M. (2023). Cilia function as calcium-mediated mechanosensors that instruct left-right asymmetry. Science 379, 71–78. 10.1126/science.abq7317 36603098 PMC9939240

[B18] Duboc V. Lepage T. (2008). A conserved role for the nodal signaling pathway in the establishment of dorso-ventral and left-right axes in deuterostomes. J. Exp. Zool. B Mol. Dev. Evol. 310, 41–53. 10.1002/jez.b.21121 16838294

[B19] Earman J. (2004). Curie's principle and spontaneous symmetry breaking. Int. Stud. Philosophy Sci. 18, 173–198. 10.1080/0269859042000311299

[B20] Essner J. J. Amack J. D. Nyholm M. K. Harris E. B. Yost H. J. (2005). Kupffer's vesicle is a ciliated organ of asymmetry in the zebrafish embryo that initiates left-right development of the brain, heart and gut. Development 132, 1247–1260. 10.1242/dev.01663 15716348

[B21] Firmino J. Rocancourt D. Saadaoui M. Moreau C. Gros J. (2016). Cell division drives epithelial cell rearrangements during gastrulation in chick. Dev. Cell. 36, 249–261. 10.1016/j.devcel.2016.01.007 26859350 PMC6485541

[B22] Fujinaga M. Baden J. M. (1991). Critical period of rat development when sidedness of asymmetric body structures is determined. Teratology 44, 453–462. 10.1002/tera.1420440411 1962290

[B23] Grande C. Patel N. H. (2009). Nodal signalling is involved in left-right asymmetry in snails. Nature 457, 1007–1011. 10.1038/nature07603 19098895 PMC2661027

[B24] Gros J. Feistel K. Viebahn C. Blum M. Tabin C. J. (2009). Cell movements at Hensen's node establish left/right asymmetric gene expression in the chick. Science 324, 941–944. 10.1126/science.1172478 19359542 PMC2993078

[B25] Hamada H. (2020). Molecular and cellular basis of left-right asymmetry in vertebrates. Proc. Jpn. Acad. Ser. B Phys. Biol. Sci. 96, 273–296. 10.2183/pjab.96.021 32788551 PMC7443379

[B26] Hashimoto M. Hamada H. (2010). Translation of anterior-posterior polarity into left-right polarity in the mouse embryo. Curr. Opin. Genet. Dev. 20, 433–437. 10.1016/j.gde.2010.04.002 20439159

[B27] Hashimoto M. Shinohara K. Wang J. Ikeuchi S. Yoshiba S. Meno C. (2010). Planar polarization of node cells determines the rotational axis of node cilia. Nat. Cell. Biol. 12, 170–176. 10.1038/ncb2020 20098415

[B28] Juan T. Geminard C. Coutelis J. B. Cerezo D. Poles S. Noselli S. (2018). Myosin1D is an evolutionarily conserved regulator of animal left-right asymmetry. Nat. Commun. 9, 1942. 10.1038/s41467-018-04284-8 29769531 PMC5955935

[B29] Kajikawa E. Horo U. Ide T. Mizuno K. Minegishi K. Hara Y. (2020). Nodal paralogues underlie distinct mechanisms for visceral left-right asymmetry in reptiles and mammals. Nat. Ecol. Evol. 4, 261–269. 10.1038/s41559-019-1072-2 31907383

[B30] Kartagener M. Stucki P. (1962). Bronchiectasis with situs inversus. Arch. Pediatr. 79, 193–207. 14454074

[B31] Katoh T. A. Omori T. Mizuno K. Sai X. Minegishi K. Ikawa Y. (2023). Immotile cilia mechanically sense the direction of fluid flow for left-right determination. Science 379, 66–71. 10.1126/science.abq8148 36603091

[B32] Kremnyov S. Henningfeld K. Viebahn C. Tsikolia N. (2018). Divergent axial morphogenesis and early shh expression in vertebrate prospective floor plate. Evodevo 9, 4. 10.1186/s13227-017-0090-x 29423139 PMC5791209

[B33] Lee H. Camuto C. M. Niehrs C. (2024). R-Spondin 2 governs xenopus left-right body axis formation by establishing an FGF signaling gradient. Nat. Commun. 15, 1003. 10.1038/s41467-024-44951-7 38307837 PMC10837206

[B34] Levin M. Johnson R. L. Stern C. D. Kuehn M. Tabin C. (1995). A molecular pathway determining left-right asymmetry in chick embryogenesis. Cell. 82, 803–814. 10.1016/0092-8674(95)90477-8 7671308

[B35] Lin C. R. Kioussi C. O'Connell S. Briata P. Szeto D. Liu F. (1999). Pitx2 regulates lung asymmetry, cardiac positioning and pituitary and tooth morphogenesis. Nature 401, 279–282. 10.1038/45803 10499586

[B36] Maerker M. Getwan M. Dowdle M. E. McSheene J. C. Gonzalez V. Pelliccia J. L. (2021). Bicc1 and dicer regulate left-right patterning through post-transcriptional control of the nodal inhibitor Dand5. Nat. Commun. 12, 5482. 10.1038/s41467-021-25464-z 34531379 PMC8446035

[B37] Manner J. (2001). Does an equivalent of the “ventral node” exist in chick embryos? A scanning electron microscopic study. Anat. Embryol. Berl. 203, 481–490. 10.1007/s004290100183 11453165

[B38] May-Simera H. L. Kai M. Hernandez V. Osborn D. P. Tada M. Beales P. L. (2010). Bbs8, together with the planar cell polarity protein Vangl2, is required to establish left-right asymmetry in zebrafish. Dev. Biol. 345, 215–225. 10.1016/j.ydbio.2010.07.013 20643117

[B39] McAlpine W. Wang K. W. Choi J. H. San M. M. McAlpine S. G. Russell J. (2018). The class I myosin MYO1D binds to lipid and protects against colitis. Dis. Model Mech. 11, dmm035923. 10.1242/dmm.035923 30279225 PMC6176994

[B40] McGrath J. Somlo S. Makova S. Tian X. Brueckner M. (2003). Two populations of node monocilia initiate left-right asymmetry in the mouse. Cell. 114, 61–73. 10.1016/s0092-8674(03)00511-7 12859898

[B41] Mendes R. V. Martins G. G. Cristovao A. M. Saude L. (2014). N-cadherin locks left-right asymmetry by ending the leftward movement of Hensen's node cells. Dev. Cell. 30, 353–360. 10.1016/j.devcel.2014.06.010 25117685

[B42] Middelkoop T. C. Garcia-Baucells J. Quintero-Cadena P. Grill S. Yazdi S. Sternberg P. W. (2021). CYK-1/Formin activation in cortical RhoA signaling centers promotes organismal left-right symmetry breaking. Proc. Natl. Acad. Sci. U. S. A. 118, e2021814118. 10.1073/pnas.2021814118 33972425 PMC8157923

[B43] Mizuno K. Shiozawa K. Katoh T. A. Minegishi K. Ide T. Ikawa Y. (2020). Role of Ca(2+) transients at the node of the mouse embryo in breaking of left-right symmetry. Sci. Adv. 6, eaba1195. 10.1126/sciadv.aba1195 32743070 PMC7375832

[B44] Naganathan S. R. Furthauer S. Nishikawa M. Julicher F. Grill S. W. (2014). Active torque generation by the actomyosin cell cortex drives left-right symmetry breaking. Elife 3, e04165. 10.7554/eLife.04165 25517077 PMC4269833

[B45] Negretti M. I. Böse N. Petri N. Kremnyov S. Tsikolia N. (2022). Nodal asymmetry and hedgehog signaling during vertebrate left–right symmetry breaking. Front. Cell. Dev. Biol. 10, 957211. 10.3389/fcell.2022.957211 36172285 PMC9511907

[B46] Nonaka S. Tanaka Y. Okada Y. Takeda S. Harada A. Kanai Y. (1998). Randomization of left-right asymmetry due to loss of nodal cilia generating leftward flow of extraembryonic fluid in mice lacking KIF3B motor protein. Cell. 95, 829–837. 10.1016/s0092-8674(00)81705-5 9865700

[B47] Nonaka S. Shiratori H. Saijoh Y. Hamada H. (2002). Determination of left-right patterning of the mouse embryo by artificial nodal flow. Nature 418, 96–99. 10.1038/nature00849 12097914

[B48] Nonaka S. Yoshiba S. Watanabe D. Ikeuchi S. Goto T. Marshall W. F. (2005). *De novo* formation of left-right asymmetry by posterior tilt of nodal cilia. PLoS Biol. 3, e268. 10.1371/journal.pbio.0030268 16035921 PMC1180513

[B49] Oki S. Kitajima K. Marques S. Belo J. A. Yokoyama T. Hamada H. (2009). Reversal of left-right asymmetry induced by aberrant nodal signaling in the node of mouse embryos. Development 136, 3917–3925. 10.1242/dev.039305 19906859

[B50] Onjiko R. M. Morris S. E. Moody S. A. Nemes P. (2016). Single-cell mass spectrometry with multi-solvent extraction identifies metabolic differences between left and right blastomeres in the 8-cell frog (xenopus) embryo. Analyst 141, 3648–3656. 10.1039/c6an00200e 27004603 PMC4899105

[B51] Onjiko R. M. Nemes P. Moody S. A. (2021). Altering metabolite distribution at xenopus cleavage stages affects left-right gene expression asymmetries. Genesis 59, e23418. 10.1002/dvg.23418 33826226 PMC8943826

[B52] Ott T. B. A. Szenker-Ravi E. Kurrle Y. Aberle O. Tisler M. Blum M. (2025). Schweickert, A MMP21 behaves as a fluid flow transported morphogen to impart laterality during development. eLife. 10.7554/eLife.104430

[B53] Otto A. Pieper T. Viebahn C. Tsikolia N. (2014). Early left-right asymmetries during axial morphogenesis in the chick embryo. Genesis 52, 614–625. 10.1002/dvg.22773 24648137

[B54] Pagan-Westphal S. M. Tabin C. J. (1998). The transfer of left-right positional information during chick embryogenesis. Cell. 93, 25–35. 10.1016/s0092-8674(00)81143-5 9546389

[B55] Petri N. Nordbrink R. Tsikolia N. Kremnyov S. (2022). Abnormal left-right organizer and laterality defects in xenopus embryos after formin inhibitor SMIFH2 treatment. PLoS One 17, e0275164. 10.1371/journal.pone.0275164 36342927 PMC9639825

[B56] Petri N. Vetrova A. Tsikolia N. Kremnyov S. (2024). Molecular anatomy of emerging xenopus left-right organizer at successive developmental stages. Dev. Dyn. 254, 950–964. 10.1002/dvdy.722 38934270 PMC12344217

[B57] Pieper T. Carpaij M. Reinermann J. Surchev L. Viebahn C. Tsikolia N. (2020). Matrix-filled microcavities in the emerging avian left-right organizer. Dev. Dyn. 249, 496–508. 10.1002/dvdy.133 31729123

[B58] Pimpale L. G. Middelkoop T. C. Mietke A. Grill S. W. (2020). Cell lineage-dependent chiral actomyosin flows drive cellular rearrangements in early *Caenorhabditis elegans* development. Elife 9, e54930. 10.7554/eLife.54930 32644039 PMC7394549

[B59] Rozbicki E. Chuai M. Karjalainen A. I. Song F. Sang H. M. Martin R. (2015). Myosin-II-mediated cell shape changes and cell intercalation contribute to primitive streak formation. Nat. Cell. Biol. 17, 397–408. 10.1038/ncb3138 25812521 PMC4886837

[B60] Saadaoui M. Rocancourt D. Roussel J. Corson F. Gros J. (2020). A tensile ring drives tissue flows to shape the gastrulating amniote embryo. Science 367, 453–458. 10.1126/science.aaw1965 31974255

[B61] Schneider I. Kreis J. Schweickert A. Blum M. Vick P. (2019). A dual function of FGF signaling in xenopus left-right axis formation. Development 146, dev173575. 10.1242/dev.173575 31036544

[B62] Schroder S. S. Tsikolia N. Weizbauer A. Hue I. Viebahn C. (2016). Paraxial nodal expression reveals a novel conserved structure of the left-right organizer in four mammalian species. Cells Tissues Organs 201, 77–87. 10.1159/000440951 26741372

[B63] Schweickert A. Vick P. Getwan M. Weber T. Schneider I. Eberhardt M. (2010). The nodal inhibitor coco is a critical target of leftward flow in xenopus. Curr. Biol. 20, 738–743. 10.1016/j.cub.2010.02.061 20381352

[B64] Shook D. R. Majer C. Keller R. (2004). Pattern and morphogenesis of presumptive superficial mesoderm in two closely related species, *Xenopus laevis* and Xenopus tropicalis. Dev. Biol. 270, 163–185. 10.1016/j.ydbio.2004.02.021 15136148

[B65] Shylo N. A. Smith S. E. Price A. J. Guo F. McClain M. Trainor P. A. (2023). Morphological changes and two nodal paralogs drive left-right asymmetry in the squamate veiled chameleon (C. Calyptratus). Front. Cell. Dev. Biol. 11, 1132166. 10.3389/fcell.2023.1132166 37113765 PMC10126504

[B66] Song H. Hu J. Chen W. Elliott G. Andre P. Gao B. (2010). Planar cell polarity breaks bilateral symmetry by controlling ciliary positioning. Nature 466, 378–382. 10.1038/nature09129 20562861 PMC3065171

[B67] Speder P. Adam G. Noselli S. (2006). Type ID unconventional myosin controls left-right asymmetry in drosophila. Nature 440, 803–807. 10.1038/nature04623 16598259

[B68] Stern C. D. (2004). Gastrulation in the chick. In: Stern C. D., editors. Gastrulation, from cells to embryo. Cold Spring Harbor, NY: Cold Spring Harbor Laboratory Press. p. 219–232.

[B69] Sulik K. Dehart D. B. Iangaki T. Carson J. L. Vrablic T. Gesteland K. (1994). Morphogenesis of the murine node and notochordal plate. Dev. Dyn. 201, 260–278. 10.1002/aja.1002010309 7881129

[B70] Supp D. M. Witte D. P. Potter S. S. Brueckner M. (1997). Mutation of an axonemal dynein affects left-right asymmetry in inversus viscerum mice. Nature 389, 963–966. 10.1038/40140 9353118 PMC1800588

[B71] Tabin C. J. Vogan K. J. (2003). A two-cilia model for vertebrate left-right axis specification. Genes Dev. 17, 1–6. 10.1101/gad.1053803 12514094

[B72] Tanaka Y. Morozumi A. Hirokawa N. (2023). Nodal flow transfers polycystin to determine mouse left-right asymmetry. Dev. Cell. 58, 1447–1461.e6. 10.1016/j.devcel.2023.06.002 37413993

[B73] Tingler M. Brugger A. Feistel K. Schweickert A. (2022). dmrt2 and myf5 link early somitogenesis to left-right axis determination in *Xenopus laevis* . Front. Cell. Dev. Biol. 10, 858272. 10.3389/fcell.2022.858272 35813209 PMC9260042

[B74] Tsikolia N. Schroder S. Schwartz P. Viebahn C. (2012). Paraxial left-sided nodal expression and the start of left-right patterning in the early chick embryo. Differentiation 84, 380–391. 10.1016/j.diff.2012.09.001 23142734

[B75] Tsikolia N. Nguyen D. T. L. Tee Y. H. (2025). Mechanisms of left-right symmetry breaking across scales. Curr. Opin. Cell. Biol. 95, 102564. 10.1016/j.ceb.2025.102564 40544609

[B76] Vandenberg L. N. Levin M. (2013). A unified model for left-right asymmetry? Comparison and synthesis of molecular models of embryonic laterality. Dev. Biol. 379, 1–15. 10.1016/j.ydbio.2013.03.021 23583583 PMC3698617

[B77] Voiculescu O. Bertocchini F. Wolpert L. Keller R. E. Stern C. D. (2007). The amniote primitive streak is defined by epithelial cell intercalation before gastrulation. Nature 449, 1049–1052. 10.1038/nature06211 17928866

[B78] Wan L. Q. Vunjak-Novakovic G. (2011). Micropatterning chiral morphogenesis. Commun. Integr. Biol. 4, 745–748. 10.4161/cib.17649 22446544 PMC3306348

[B79] Xu J. Van Keymeulen A. Wakida N. M. Carlton P. Berns M. W. Bourne H. R. (2007). Polarity reveals intrinsic cell chirality. Proc. Natl. Acad. Sci. U. S. A. 104, 9296–9300. 10.1073/pnas.0703153104 17517645 PMC1890488

[B80] Yamanaka H. Kondo S. (2015). Rotating pigment cells exhibit an intrinsic chirality. Genes cells. 20, 29–35. 10.1111/gtc.12194 25345494

