# Supplementary figures and images for: Left-right symmetry breaking: learning from the chicken
Source: Front Cell Dev Biol. 2025 Sep 24;13:1672263. doi: 10.3389/fcell.2025.1672263 (PMC12504268; doi:10.3389/fcell.2025.1672263)

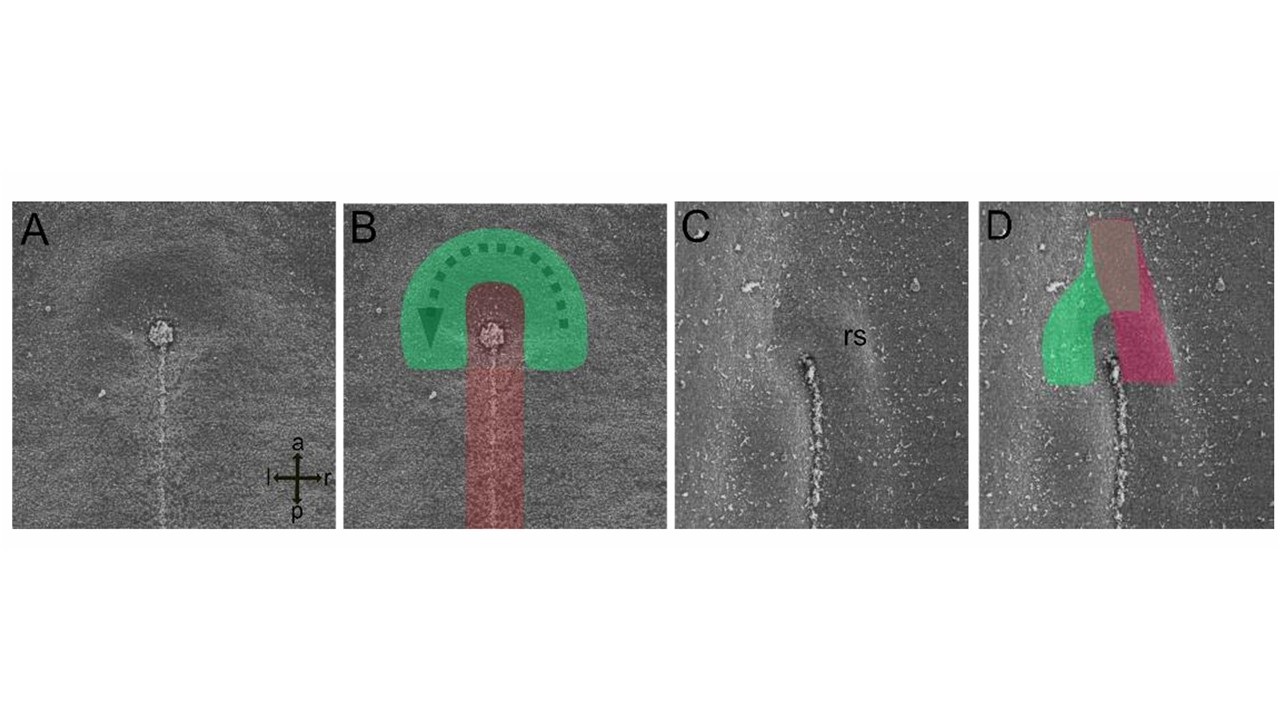

Supplement: Supplementary file 1 [file Image1.jpeg]
